# Erratum for Benler et al., “Cargo Genes of Tn*7*-Like Transposons Comprise an Enormous Diversity of Defense Systems, Mobile Genetic Elements, and Antibiotic Resistance Genes”

**DOI:** 10.1128/mbio.00257-22

**Published:** 2022-03-01

**Authors:** Sean Benler, Guilhem Faure, Han-Altae Tran, Sergey Shmakov, Feng Zhang, Eugene Koonin

**Affiliations:** a National Center for Biotechnology Information, National Library of Medicine, Bethesda, Maryland, USA; b Broad Institute of MIT and Harvard, Cambridge, Massachusetts, USA; c HHMI, Massachusetts Institute of Technology, Cambridge, Massachusetts, USA; d McGovern Institute for Brain Research, Massachusetts Institute of Technology, Cambridge, Massachusetts, USA; e Department of Brain and Cognitive Sciences, Massachusetts Institute of Technology, Cambridge, Massachusetts, USA; f Department of Biological Engineering, Massachusetts Institute of Technology, Cambridge, Massachusetts, USA

## ERRATUM

Volume 12, no. 6, e02938-21, 2021, https://doi.org/10.1128/mBio.02938-21. Feng Zhang’s last name was spelled incorrectly in the original article. The byline should appear as shown in this erratum.

